# Usability Testing of a Mobile Health Intervention to Address Acute Care Needs after Sexual Assault

**DOI:** 10.3390/ijerph16173088

**Published:** 2019-08-25

**Authors:** Amanda K. Gilmore, Tatiana M. Davidson, Ruschelle M. Leone, Lauren B. Wray, Daniel W. Oesterle, Christine K. Hahn, Julianne C. Flanagan, Kathleen Gill-Hopple, Ron Acierno

**Affiliations:** 1 College of Nursing, Medical University of South Carolina, Charleston, SC 29425, USA; 2 Department of Psychiatry & Behavioral Sciences, Medical University of South Carolina, Charleston, SC 29425, USA; 3 Forensic Nursing Services, Medical University of South Carolina, Charleston, SC 29425, USA; 4 Ralph H. Johnson VA Medical Center, Charleston, SC 29425, USA; 5 Department of Psychiatry, University of Texas Health Science Center, Houston, TX 77030, USA

**Keywords:** sexual assault, *m*Health, usability

## Abstract

Sexual assault is associated with a range of poor mental health outcomes. To enhance access to care by this population, technology-based mental health interventions have been implemented in the emergency room; however, more accessible and easily disseminated interventions are needed. The aim of the present study was to test the usability of a mobile health intervention targeting alcohol and drug misuse, suicide prevention, posttraumatic stress symptoms, coping skills, and referral to formal assistance for individuals who have experienced sexual assault. Feedback on the usability of the intervention was collected from individuals who received a sexual assault medical forensic examination (*n* = 13), and feedback on the usability and likelihood of recommending the application was collected from community providers (*n* = 25). Thematic analysis was used to describe qualitative data. Content themes related to aesthetics, usability, barriers to resources, and likes/dislikes about the intervention arose from interviews following the intervention. Participants found the intervention to be user friendly and endorsed more likes than dislikes. Providers rated the intervention as being helpful and would recommend it to survivors of sexual assault. Findings suggest that the intervention is usable and fit for future effectiveness testing, filling an important gap in treatment for individuals who experience sexual assault.

## 1. Addressing Barriers to Acute Care after Sexual Assault: Usability of a Mobile Health Intervention

Sexual assault is defined as a nonconsensual sexual experience that can range from sexual contact to penetration. Sexual assault is common; the National Intimate Partner and Sexual Violence Survey found that 13.1% to 46.1% of women experience nonconsensual penetration (i.e., rape) and 25.9% to 58.0% experience sexual assault other than rape during their lifetime, with prevalence differing based on sexual orientation [1]. There are significant health costs associated with sexual assault, and the US government pays an estimated $1 trillion of the total estimated $3.1 trillion of lifetime costs accrued by sexual assault victims in this country [2]. Sexual assault can result in long-term mental health effects, including alcohol and drug use disorders, suicidality, posttraumatic stress disorder (PTSD), and depressive disorders [3]. Although most who experience sexual assault evince a decline in initial post-assault symptoms within the first few months following an assault [4], for many, these symptoms are pervasive and long-lasting if untreated. Therefore, it is essential to provide individuals who have experienced sexual assault with preventative services when and where they are already accessing care. A sexual assault medical forensic examination (SAMFE) is one place where individuals are accessing care within an acute time frame after the sexual assault, typically within 72 to 120 h [5]. There are several systemic (e.g., access to services), logistical (e.g., financial constraints), and attitudinal (e.g., stigma, confidentiality concerns) barriers to accessing healthcare services immediately after sexual assault [6,7,8]. Therefore, it may be beneficial to provide mental health information and coping skills to individuals in this acute period using a mobile health (*m*Health) application after receiving a SAMFE. The current study examined the usability of a *m*Health application targeting common post-sexual assault mental and physical health concerns, including alcohol and drug use disorders, depressive symptoms, and acute stress/posttraumatic stress symptoms. 

### 1.1. Barriers to Follow-Up Healthcare after a SAMFE

Despite the availability of evidence-based treatment for common symptoms after sexual assault, 60% to 72% of individuals who receive a SAMFE do not receive recommended follow-up mental and medical health services [9,10,11]. Further, the overwhelming majority of male and female adult sexual assault survivors deny ever receiving mental health services related to their experience of sexual assault [12,13]. Common systemic and logistical barriers to receiving healthcare by the general population are also experienced by those who have received a SAMFE, including lack of child care, transportation difficulties, and limited access to services [6]. Common beliefs that are reported as post-sexual assault barriers to use of healthcare include perceived stigma, internalized self-blame, and concerns about confidentiality [7,8]. Moreover, compelling evidence indicates that the “desire to handle the problem on one’s own” is the most common attitudinal barrier to seeking treatment—73% in the U.S.—among adults with perceived need for mental health treatment [14,15], and has been a reported barrier to post-sexual assault healthcare among men [16,17].

### 1.2. mHealth as a Viable Solution to Address Key Barriers

Technology-based resources have potential to overcome many of the common barriers to accessing follow-up healthcare, particularly mental health care, after a SAMFE. For example, *m*Health (e.g., smartphone, tablet, web) interventions can be accessed instantly (barrier: scheduling, time commitment), freely (barrier: cost), privately (barrier: stigma), and almost anywhere (barriers: logistical [time commitment travel, parking, waiting room, travel costs] and transportation), and can be tailored to the needs of the user (barriers: cultural values, language). Further, self-help solutions available via technology may be ideally suited to address the key barrier of wanting to handle the problem oneself [14]. 

Over 75% of US adults own a smartphone. Hispanic (71%) and Black (70%) adults are slightly more likely to own a smartphone than White adults (61%), and rates of smartphone ownership are 50 and 71% for low-income and poverty level families, respectively. In 2015, roughly 78% of the US population had a unique mobile subscription and smartphone adoption was over 75%. Over 70% of adults within the U.S. seek health-related information online [18], and nearly two-thirds of mobile phone users have downloaded a mobile application (app) at some point in their lives. These data are encouraging because they suggest mobile interventions, many of which are effective in producing behavior change in physical and/or mental health conditions, are likely to be used by diverse populations and may be readily integrated to reduce common barriers to receipt of services. 

### 1.3. Previous Research on Technology-Based Interventions in This Population

Technology-based interventions within the context of a SAMFE are not new to the field of prevention science. Resnick and colleagues developed a Prevention for Post Rape Stress video that provides psychoeducation in the emergency department during a SAMFE and has been effective at reducing post-sexual assault substance use and mental health symptoms among those with prior sexual assault histories and those who used substances prior to the assault [19,20,21,22]. Providing mindfulness techniques in a video to individuals during the SAMFE, compared to treatment as usual, was effective at reducing post-sexual assault prescription opioid misuse 1.5 months after the assault among women with a prior history of sexual assault [23]. However, not all technology-based programs have been successful. One study examined the feasibility of a four-week text messaging program led by sexual assault nurse examiners and found that the majority of individuals did not complete the program [24]. The low participation in the program may suggest that not all individuals are interested in receiving healthcare information from a healthcare professional after the SAMFE, perhaps due to similar barriers related to accessing this healthcare in general among this population. Nonetheless, using *m*Health technology among individuals with recent sexual assault may reduce the barriers to seeking follow up care and the potential negative post-sexual assault mental health symptoms. To our knowledge, no study to date has examined the usability of a *m*Health application for this population. Due to the potential wide-reach of *m*Health applications and low cost to disseminate, this may be a viable next step to prevent post-sexual assault mental health symptoms.

### 1.4. Current Study

Due to the significant barriers associated with accessing post-sexual assault healthcare, in addition to barriers associated with accessing healthcare among underrepresented minorities that are at high-risk for experiencing sexual assault, the current study assessed the usability of a mobile health application for individuals who experienced recent sexual assault, *SC-Safe*, using a mixed method approach. We also assessed initial usability and likelihood of recommending this application to individuals who recently experienced sexual assault among community providers. 

## 2. Methods

### 2.1. Mobile Health Structure

**Figure 1:**
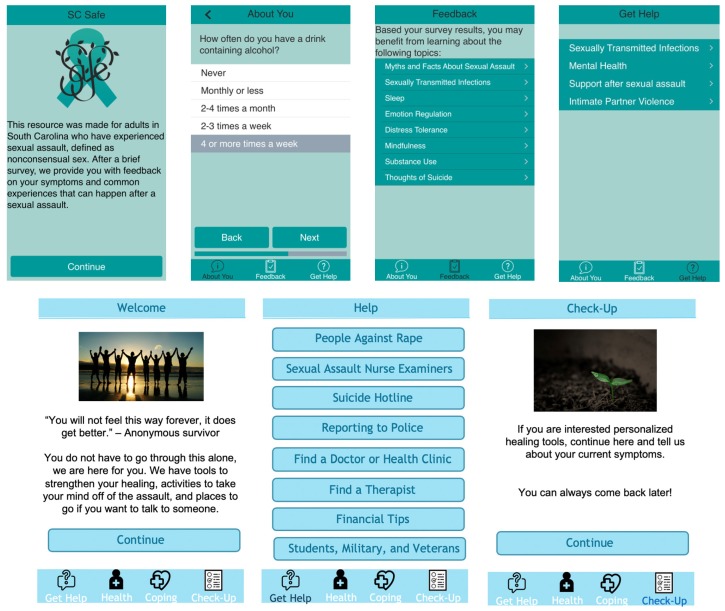
Initial prototype tested in the current study. Revised prototype based on feedback from usability testing.

*SC-Safe* is a resource designed for individuals over the age of 18 residing in South Carolina who have experienced sexual assault. It was designed by the first and second author to address a gap in clinical services after recent sexual assault. Therefore, the application was modeled after services that were delivered in a clinical setting, and included screening and brief intervention using evidence-based practices. The current study provides a description of the first feedback that the research team received on the content from individuals who experienced sexual assault. *SC-Safe* has five major intervention modules and a referral to treatment component: (1) alcohol and substance use, (2) suicide prevention, (3) posttraumatic stress and depressive symptoms, (4) adaptive coping skills including mindfulness, distress tolerance, and emotion regulation, (5) physical health including sexual health and sleep, and (6) referral to formal assistance through various community resources. Upon completion of a brief survey, users receive a recommendation about which modules to use based on their responses. Modules consist of personalized psychoeducation and skills in the form of brief descriptive text and interactive learning exercises. Users can access all of the modules if they prefer. Each module is described in greater detail below. See Figure 1 for the initial application that was tested in the current study as well as a revised draft based on feedback from participants.

*Alcohol and Drug Use.* Users receive education regarding short-term and long-term consequences of alcohol and drug use. This information includes commonly reported reasons for using alcohol and drugs post-sexual assault, as well as alternatives to coping through substance use. A harm reduction approach [25] was taken for alcohol and drug use to encourage use within the recommended limits based on gender. 

*Suicide Prevention.* This module provides the user with information about suicide risk factors, warning signs, myths and truths about suicide, and the Suicide Prevention Lifeline and services finder.

*Posttraumatic Stress and Depressive Symptoms.* Under the posttraumatic stress component, users are provided with education about common responses to sexual assault (e.g., fear and anxiety) as well as education and rationale supporting the benefits of in-vivo and written exposure, both active components of evidence-supported treatment modalities for sexual assault survivors [26,27]. For the in-vivo exposures, users are guided through making a fear hierarchy and are given detailed instructions for completing writing exposures on a weekly basis. Similarly, a behavioral activation approach [28] consistent with manualized, evidence-supported treatment addresses depressive symptoms and users are provided with activity ideas to choose from and encouraged to create an activities schedule.

*General Adaptive Coping Skills.* Users are given three main components designed to promote emotional health and emotional recovery, including emotion regulation, distress tolerance and mindfulness activities (e.g., breathing exercises, meditation, and other activities to focus on the current moment) [29]. Each of these components contains interactive features (e.g., flip cards) to assist users in learning common reactions following assault and different adaptive coping strategies to manage difficult emotions effectively. 

*Physical Health.* Users are provided with information regarding the recommended follow-up medical visits from the Centers for Disease Control [30]. Specifically, it is recommended that medical follow-up care be accessed 1, 3, and 6 months after the sexual assault to receive testing for sexually transmitted infections as well as any medical care or treatment that is needed. Given that sleep disruption is common after experiencing a sexual assault, sleep hygiene was also provided. 

*Referral to Formal Assistance.* Users are provided with education about testing for sexually transmitted infections, “best practice” mental health interventions, support after sexual assault, and intimate partner violence. Users had access to resources that link directly to local, regional and national treatment settings and organizations that provide additional information. 

### 2.2. Usability Testing Overview

The purpose of usability testing is to obtain objective metrics and identify opportunities to strengthen the quality of a product. Common usability problems include issues that prevent task completion, take the user off course from the task, create confusion, and/or decrease satisfaction with the product; more specific examples include performing the wrong action, misinterpreting something, and not understanding the navigation [31]. Several studies have examined how usability testing can improve user experiences with direct-to-consumer online products targeting health conditions [32,33,34,35,36] and formal usability testing generally yields a high return on investment with regard to products that required tremendous costs in time, effort, and funding to develop [31].

### 2.3. Procedure

Potential participants were identified after receiving a SAMFE within 120 h of a sexual assault. The protocol of SAMFE treatment included the nurse examiner offering the patient the opportunity to participate in research studies. The patient was provided a form to accept or decline follow-up contact by a researcher. Only those who consented to being contacted were invited to participate in the study. Potential participants were contacted by letters and phone calls to participate in a study to obtain feedback on a *m*Health application for people who have recently experienced sexual assault. Participants were informed that the self-help app (a) was designed for individuals aged 18 and older in South Carolina who have experienced sexual assault; (b) would target alcohol and substance use, suicidal ideation, posttraumatic stress and mood; (c) would provide coping skills education; and (d) would provide a list of local and national resources. 

Following informed consent, participants were then asked to download *SC-Safe* from the app store on their phone, while an interviewer observed their use of the app. This stage of testing involved an introductory observation phase during which the participant was encouraged to freely navigate the app, followed by an evaluation phase using a cognitive interviewing approach [37]. The interviews were audio recorded and transcribed. Interviewers were trained to ask all relevant usability questions linked to the actions of the participant but were granted the flexibility to add or modify questions as needed to clarify participants’ responses and to follow up on unanticipated comments and/or questions. Once the participant finished using the application, the interviewer administered a final set of semi-structured interview questions to learn more about participants’ experiences with *SC-Safe*. Interviewers were permitted the flexibility to probe and ask relevant follow-up questions as needed. This approach was selected because it solicits the same “core” information from all participants in a systematic manner while also benefiting from the strengths of a qualitative interview approach, which values individual perspectives. In each content area, participants were asked opinions about the look of each page, what they liked and disliked about the page, and how we might make each page more interesting. Further, participants were asked more generally what they thought about *SC-Safe*, what they liked and disliked about *SC-Safe*, whether it would be useful to tell recent victims about *SC-Safe* during the SAMFE, if they thought *SC-Safe* would be useful (why or why not), if they thought other people would like using *SC-Safe* (why or why not), and if other people would use SC-Safe (why or why not). 

Usability testing and follow-up interviews were conducted in-person or through teleconferencing, according to participant preference, and lasted 45 to 60 min. Participants were reimbursed $50 for their participation. All study procedures were approved by the Institutional Review Board of the hospital (Pro00068707).

For service providers, feedback was received on *SC-Safe* within the context of a group presentation of the application rather than having providers download the application. Providers were asked to complete a brief survey regarding *SC-Safe* to be used to improve the application and interviews were not conducted.

### 2.4. Participants

Participants were (1) individuals who experienced sexual assault and (2) providers who serve individuals who experience recent sexual assault. Individuals who experienced sexual assault were recruited from a local hospital in South Carolina if they experienced a sexual assault and received a SAMFE. Participants were recruited from a database of individuals who had received a SAMFE in the past two years. A total of 149 potential individuals were recruited to participate in the study, and 13 participated (11.46%). This participation rate is consistent with other research from the emergency department. A total of 13 participants completed the usability interview (*M_age_* = 28.00; *SD* = 7.58). As displayed in Table 1, most participants identified as White, female, and were single. Approximately two-thirds of participants were not in college (*n* = 10) and had medical insurance (*n* = 10). The length of time since the sexual assault varied among participants (*M*_months_ = 12.09; *SD*_months_ = 7.13). The assaults were perpetrated by an acquaintance (*n* = 7), stranger (*n* = 5), and partner (*n* = 1), respectively. Providers (*n* = 25) who serve individuals who experience recent sexual assault were recruited to complete an anonymous survey about the *m*Health application. 

Providers were recruited during a local Sexual Assault Response Team meeting and their participation was voluntary. A presentation was given about the application in a group format by the first author.

### 2.5. Measures

*Sexual Assault Characteristics.* Participants were asked to indicate if the perpetrator was “someone you didn’t know before”, a “casual acquaintance”, or “a partner (dating partner or spouse)”. This question was used to assess who the perpetrator of the assault was using the three most common perpetrators. Participants were also asked “Were you passed out or unable to consent or stop what was happening due to the effects of alcohol or drugs?” with response options being yes or no. This question was adapted for clinical purposes and was adapted from the Sexual Experiences Survey [38]. 

*Current Mental Health and Substance Use Symptoms (Included in SC-Safe).* To assess for depressed mood and anhedonia over the past two weeks, The Patient Health Questionnaire, which has strong validity and reliability (PHQ-2) [39], was used. Scores of these items ranged from 0 to 2, with 0 (“not at all”) to 3 (“nearly every day”), with total scores of 3+ indicating depressed mood. Posttraumatic stress symptoms (PTSD) were assessed using the PTSD Checklist (PCL-5) [40], which had high internal consistency (α = 0.96) and convergent and discriminant validity in previous research [41,42]. This measure includes 20 items to assess the degree (or severity) of distress by specific posttraumatic stress symptoms over the past month with responses ranging from 1 (*not at all*) to 5 (*extremely*). Scores of 33 or more indicate clinical levels of PTSD symptoms [41,42]. Alcohol misuse was assessed using the Alcohol Use Disorders Identification Test (AUDIT-C) [43]. This brief screening tool has 3 items assessing for frequency and quantity of alcohol use. Individual response items range from 0 to 4 and total scores range from 0 to 12. Scores of 3 for females and 4 for males indicate clinical levels of alcohol misuse [44,45].

*Provider Experiences and Feedback.* Providers completed a 3-item survey about their experiences working with individuals who have experienced recent sexual assault. This included information regarding weekly hours spent on sexual assault issues, weekly hours spent providing direct services to sexual assault victims, and type of services provided (i.e., judicial, behavioral health, physical health, education). Participants also completed the following questions regarding *SC-Safe*: “How helpful do you think *SC-Safe* (app) would be for individuals who have experienced sexual assault” with answer choices ranging from 1 (*Not at all helpful)* to 7 (*Very helpful*) and “How likely would you be to recommend *SC-Safe* to individuals who have experienced sexual assault” with answer choices ranging from 1 (*Not at all likely*) to 7 (*Very likely).*

### 2.6. Data Analytic Plan

*Quantitative Approach.* Descriptive statistics were used to examine the characteristics of the participants and provider feedback regarding *SC-Safe*.

*Qualitative Approach.* The qualitative approach chosen for this project is thematic analysis [46], which was guided by constructivist grounded theory [47,48] for coding the data. This is an approach that acknowledges the researchers’ prior knowledge and influence in the process, and supports and provides guidelines for building a conceptual framework to understand the interrelations (e.g., what and how) between constructs [49]. First, a content analysis of responses was conducted through multiple close readings of transcriptions by two independent coders, including the first author. Each coder generated an independent list of thematic categories and subcategories based on their review of the data. These themes were then further developed and ordered by the first author and reviewed. The authors then met in a consensus conference to discuss the categories, resolve questions, and refine the thematic categories prior to developing the final thematic categories. This thematic analysis method was guided by constructivist grounded theory such that an inductive approach was used to generate hypotheses from the qualitative data, and themes from earlier interviews shaped continued data collection until thematic saturation was met [49]. Recent projects evaluating pilot data and individual interview feedback for adapting interventions have used similar analytic approaches to qualitative research [50,51,52]. Kappas above 0.60 are rated as reliable [53]. Coding strategies indicated high levels of inter-rater agreement with an average kappa of 0.90.

## 3. Results

### 3.1. Mental Health and Substance Use Sample Descriptives 

Sample descriptive statistics are presented in Table 1. A total of 38.5% participants indicated that they experienced the sexual assault within one year of participating in the study and the majority of participants (53.8%) indicated that the sexual assault was perpetrated by an acquaintance (see Table 1 for more details).

On average, participants did not report depressed mood (PHQ-2: *M* = 2.31; *SD* = 2.36). A total of 5 (38.4%) screened positive for clinical levels of depression. Participants reported modest PTSD symptoms (PCL-5: *M* = 22.15; *SD* = 21.52). A total of 4 (30.8%) screened positive for clinical levels of PTSD. The average severity of alcohol problems was moderate in this sample (AUDIT-C: *M* = 4.23; *SD* = 2.89). A total of 10 participants screened positive for engaging in hazardous drinking or having an active alcohol use disorders (76.9%).

### 3.2. Qualitative Results

While conducting individual interviews, we gained valuable insight about the potential usefulness and helpfulness of *SC-Safe* for individuals who experienced recent sexual assault. Recall that no interviews were conducted with providers. The final thematic categories are presented in Table 2 and resulted in four main domains: (1) Aesthetics and Usability (opinions for improving look and feel and use), (2) Barriers to Resources (challenges to accessing available resources following assault), (3) Opinions about *SC-Safe* (participant likes and dislikes) and (4) Recommendations for *SC-Safe* (recommendations for improving the application).

*Domain 1: Aesthetics and Usability.* The majority of participants who had completed a SAMFE (75%) felt that the application looked nice, simple and was not overwhelming. About half of participants reported that the layout and color choices allowed for more privacy when using the application. For example, one participant [Female, age 38, White/Non-Hispanic] stated:

I mean it’s a really—it’s pretty basic and that’s what I like about it that it’s not busy, it’s basic and also too it’s kind of sort of discreet as well. So, if somebody else does look your phone, they don’t know immediately.

Another participant [Female, age 31, White/Ethnicity Unknown] noted:

I think neutral is good. You know, again if this is someone that has hurt you and they are close by, I mean you don’t want them to know that this is some kind of reporting or anything like that. You want it to be kind of incognito, so I think I mean I like it.

Other participants felt that the aesthetics needed updating, including using brighter colors and increasing the font size, and including inspirational images. With regard to usability, several participants expressed that it was challenging to navigate through different sections of the app. Recommendations for improvement included making the navigation functions standard across modules (e.g., swiping or using back and forward arrows) and clearly indicating the purpose of each navigation icons. 

*Domain 2: Barriers to Resources.* All participants (100%) expressed experiencing logistical or attitudinal barriers to accessing and utilizing available services (e.g., support groups, STI testing, counseling) following the SAMFE. For example, the majority of participants stated that not knowing where to go for services, concerns about cost and lack of insurance, distance from provider, and long-wait times were barriers to service use. One participant [Female, age 38, White/Non-Hispanic] expressed:

The thing honestly that kept me from going to my regular appointments afterwards was just the time because, you know, geographically I was pretty far out of there. So, to get to my appointment, have the appointment and get home, it took three, three and a half hours out of my day. And what if you’re working on job, it’s kind of difficult to—I mean you can sneak out for an hour, an hour and a half but I mean it’s kind of hard to carve out three hours in a day.

Another participant [Female, age 23, White/Non-Hispanic] noted:

Just being aware of the resources like, the ignorance of the resources. And even if they’re aware of the resources, the ignorance of the fact that like, a lot of these are not very cost preventative. They’ll work with you because they are a victim of a crime.

Attitudinal barriers, including denial, embarrassment over having to be examined, not wanting to talk with others about the assault, and fear of how others (e.g., family, friends, media) would react were noted barriers to service utilization. For example, one participant [Female, age 19, White/Non-Hispanic] expressed:

I mean not wanting to talk about that, that’s a big thing. Maybe feeling like people won’t believe you that’s a pretty big things for me at least. I guess and some people don’t really want to come forward and talk to their family about it. So, I guess how your family might react, that can be a pretty big thing.

Another participant [Female, age 38, White/Non-Hispanic] expressed:

You know, just sometimes I just I don’t want to talk about it anymore. I have talked it, I’ve gone through it, I don’t want to have to keep reliving it week after week after week for the rest of my life or six months or a year however long it takes. You know, so that’s a big emotional barrier. You know, some people like myself included, you know, I’ve had an experience. I’ve done like the more intensive therapy and, you know, it’s almost a year out now for me and I have got a life that I have to get back to.

*Domain 3: Opinions about SC-Safe.* With regard to the education module, participants felt that the information provided was helpful in addressing several myths following sexual assault, providing education and reminders about the importance of following through with STI examinations, and letting them know where they can access for information and/or potential next steps. For example, one participant [Male, age 23, White/Non-Hispanic] stated:

One of the things I really like seeing was the myth [dispelling the myth was presented in the content] “Men can’t be sexually assaulted” you know. What making perpetuate is that, I was – one of my base issues going to the group is, guys are really underrepresented in this area. It’s been really hard to find support systems just because I am a guy.

Another participant [Female, age 45, White/Non-Hispanic] expressed:

So, if they’re just looking to see if this really happened to them, it’s useful information or ‘does [this] happen to me’, let me see where I can get more information, you know, what do I do, what’s my next step? Very true, very true. People don’t get that enough. The perpetrator is always 100% responsible. People don’t know that enough. So, it’s just very well put together. It’s very well thought out.

Similarly, participants responded favorably to the emotional and behavioral health modules on alcohol and substance use, suicide prevention, posttraumatic stress and depressive symptoms. Participants expressed that the modules provided useful and relevant information, helped to de-stigmatize mental health disorders, and provided helpful tips for monitoring symptoms. For example, one participant [Female, age 23, White/Non-Hispanic] stated:

With suicide already being stigmatized the way it is, and communication about suicide being stigmatized the way it is, I would want to know that like, it’s okay to talk about this and it’s okay if this is what you’re feeling like.

When asked for impressions about the PTSD component, one participant [Female, age 20, White/Hispanic] expressed:

They still have mindset of, you can get over it, it’s not that big a deal. But it’s that deep-rooted fear through all of us rest of our lives. So, a lot of people are just going to ignore and I think that’s something that needs to be addressed and considered more than it is. And then the fears that come with the PTSD like flashbacks, nightmares, stuff like that. Yeah there is hope for that and just showing that there is something you can do about it.

Another participant [Female, age 45, White/Non-Hispanic] stated:

People don’t always know how to constructively express their selves. And they resort to drugs and alcohol. And that isn’t the way, that always makes it worse. So, if you got something like this and you said immediately, if you fail on this do this or something similar to do this. Take a walk, call neighbor, you know. They have the information right there, just they have to—they just have to get the information, the right information, not to any drugs or alcohol. So, a lot of people, that’s what people do. That’s very important.

Finally, with regard to the general coping skills module, participants felt that emotional regulation, distress tolerance and mindfulness were the most useful components in assisting to manage distress associated with assault. For example, one participant [Female, age 45, White/Non-Hispanic] expressed:

This one is the best one, because it’s says, “you may experience, even though you don’t do anything wrong, but you may experience of these too even though you didn’t do anything wrong and it’s okay to have these—it’s okay to have all these feelings. It just what you do with it”.

Another participant [Female, age 20, White/Hispanic] stated:

Stress is something that I feel it’s close with a lot of people especially people that don’t have experience this and people that are actually, you know, that are going through something, not only this but something really traumatic. So, that’s really something good to read and something that you can kind of read, kind of figure out how to control it and how to settle down and just kind of relax after going through something and you’re not exactly sure how to cope with.

When asked to share reactions on the mindfulness component, one participant [Female, age 23, White/Non-Hispanic] noted:

I do appreciate that mindfulness has its own little bar because I do feel like mindfulness is something that a lot of people aren’t aware of. They aren’t aware of the benefits, they aren’t aware of how to be mindful. Mindfulness for me is one of my go-to coping mechanisms, being aware of everything in my surrounding area especially if I’m triggered by sight, sight by location specifically. If I can pay attention to something else that’s in that area, I can distract myself from the triggering event. And I like that you break down the different types of mindfulness because, yes there is mindfulness through your senses but there’s also mindfulness in an activity.

When asked about other considerations to take into account for the development of *SC-Safe*, one participant [Gender-Nonbinary, 21, White/Non-Hispanic] stated:

Bring identity into it [SC-Safe] as much as possible. I think just saying anything about specific identities like race/ethnicity, gender and sexual orientation, included or intersected with homelessness—or just anything like that that people might have questions about beyond the general facts that you’re giving. Because that information is really useful, because people might say ‘what about this?’ and that info would be really helpful to have—especially if there has been research done already like for the group in question you could say ‘people in this group are more likely to experience something like that’.

### 3.3. Descriptive Results for Service Providers

For individuals who provide services to those who have experienced sexual assault, the current study recruited a wide variety of different service providers. Providers worked in advocacy (*n* = 11), the judicial system (*n* = 5), behavioral health (*n* = 3), law enforcement (*n* = 2), and medical providers (*n* = 3). On average, providers worked 27.19 h per week (*SD* = 16.01, *Range* = 0–50) on issues related to sexual assault and 12.22 h per week (*SD* = 13.38, *Range* = 0–40) with individuals who have experienced sexual assault. The intervention was rated by providers as being very helpful for individuals who have experienced a sexual assault (*M* = 6.13, *SD* = 0.92). Providers reported that they would be likely to recommend it to individuals (*M* = 6.35, *SD* = 0.94).

## 4. Discussion

The current study presented initial usability findings of *SC-Safe*, a *m*Health application for post-sexual assault mental health, from both individuals who received a SAMFE and providers who work with individuals who have experienced sexual assault. Similar to previous research [6,7,8], participants in this study indicated experiencing significant barriers to receiving services after sexual assault. Therefore, *SC-Safe* may be one way to address perceived barriers to accessing follow-up care given that it can be freely accessed any time that is convenient, and provides information in a manner that is trauma-informed and addresses stigma-related concerns. 

Overwhelmingly, the participants indicated that *SC-Safe* would be a helpful resource for individuals who experience sexual assault due to the pervasive barriers that these individuals face to accessing care. Providers indicated that they believed *SC-Safe* would be helpful for individuals who experienced recent sexual assault and that they would recommend it if it were freely available. Further, participants who received a SAMFE provided useful feedback regarding the usability of the application. Regarding the aesthetics and usability, participants indicated that *SC-Safe* was simple and not overwhelming and the layout allowed for privacy. Suggestions for improvement included to increase the color brightness and font size and to make navigation functions clear and uniform throughout the application. Participants provided useful feedback regarding the content of *SC-Safe*. Specifically, they indicated that the modules on behavioral health topics (i.e., substance use, suicide, posttraumatic stress disorder, depression) were informative and helpful and that they specifically enjoyed the general coping skills (e.g., mindfulness, emotion regulation) that would be helpful even if one is not experiencing clinically significant levels of mental health symptoms. Overall, the findings suggested that the usability of *SC-Safe* was successful and it would be useful to examine the efficacy of this application.

Although the participants indicated that *SC-Safe* was discreet, additional precautions may be needed to protect individuals if they experienced sexual assault in the context of intimate partner violence. Given that experiencing sexual assault in the context of intimate partner violence decreases the likelihood of engaging in follow-up services [10], it is imperative to address the service utilization gap that exists with these individuals and the unique concerns that exist for safety. 

## 5. Strengths and Limitations

The current study included a small sample of individuals to assess initial usability of *SC-Safe*. Future work is needed to understand the post-SAMFE healthcare needs of men, sexual and gender minorities, and different racial/ethnic groups. Future work is also needed to assess if there are differences in usability based on demographic characteristics and time since the most recent sexual assault. It was not possible to examine differences in the current study due to the small sample size, therefore, more work is needed. It is possible that individuals who experienced sexual assault within days and weeks of the sexual assault may have different feedback than those who experienced the sexual assault more than one year ago. Due to the high rates of sexual assault among those who identify as LGBTQIA+ [54] or belong to a racial/ethnic minority group [55], it is important to consider unique barriers to receiving healthcare among these underserved populations as well. For racial/ethnic minority groups, societal (e.g., language, cultural values) and organizational (e.g., lack of insurance, lack of documentation) barriers may even further limit access to several treatment options [56]. Among the LGBTQIA+ individuals, stigma, reluctance to disclose sexual identity, and insufficient number of culturally competent providers are significant treatment access barriers [57,58]. Prior to testing the efficacy of *SC-Safe*, it is crucial to receive feedback from sexual, gender, racial, and ethnic minority groups that are at high risk for sexual assault and that have additional barriers to accessing healthcare services. This application did not assess for human trafficking risk, which would be beneficial to add in future iterations of the application. Due to the many barriers to seeking services related to sexual assault, as well as compounding barriers for sub-populations that are at high risk for experiencing sexual assault, innovative approaches to service delivery, like *SC-Safe* are urgently needed, but further testing within these populations is urgently needed. It would be helpful for future research to integrate personalized insurance, medical costs, and applications for victim compensation to cover medically related costs, if eligible, into *SC-Safe*.

Although we observed positive feedback on the usability of this intervention, results are preliminary and further research is needed. It is typical for interviewers to be present during usability testing; however, it is possible that the presence of the researcher may have inhibited participants’ responses. Therefore, it may be useful to assess usability when there is no researcher present through self-report as well. Future research will include a more in-depth analysis of usability questionnaires, however, at the current stage of this program development we were interested in receiving more open-ended feedback to inform the second iteration of the program. Usability findings do not provide any indication of efficacy. Although evidence-based practices were integrated into *SC-Safe*, more work is needed to determine the efficacy of the application. Despite this, the strength of this study lies in the use of multiple evaluative methods to assess for usability, a needed first step in intervention development. Interviews from both individuals who received a SAMFE and providers who frequently work with this population allowed for rich feedback from multiple perspectives. In particular, quantitative results were based on perspectives from providers working in advocacy, behavioral health, the judicial system, and law enforcement, which suggest various disciplines involved in sexual assault response services support the need for future implementation of the intervention. Additionally, feedback from individuals who have experienced sexual assault will allow us to modify the intervention to more directly assess concerns following sexual assault. 

## 6. Conclusions

The current study assessed the initial usability of *SC-Safe* and findings suggest that the application is usable and fit for future effectiveness testing, filling an important gap in treatment for individuals who experience sexual assault. Future work should assess the efficacy of *SC-Safe* among a diverse group of individuals who experienced recent sexual assault prior to disseminating this intervention to the general population. Although *SC-Safe* was created initially for individuals who experienced sexual assault within the state of South Carolina, this application has the potential to address the gap of post-sexual assault healthcare needs of individuals who experience recent sexual assault nationwide.

## Figures and Tables

**Table 1:** Demographic information and descriptive statistics of individuals who experienced sexual assault.

Variables	*n*	%
Total Sample		
Race		
White	13	100
Ethnicity ^1^		
Non-Hispanic/Latina	10	
Hispanic/Latina	2	15.4
Gender		
Female	11	84.6
Male	1	7.7
Other ^2^	1	7.7
Marital Status		
Single	7	53.8
Dating	4	30.8
Serious Relationship	2	15.4
Married	1	7.7
Divorced	0	0
Student Status		
Not current a student	10	76.9
Currently a student	3	23.1
Active Duty Member		
No	12	92.3
Yes	1	7.7
Insurance Status		
Insurance	10	76.9
No Insurance	3	23.1
Time Since Sexual Assault Occurred ^1^		
2–4 weeks	1	7.7
1–6 months	2	15.4
6–12 months	2	15.4
12–24 months	6	42.2
Assaults that Involved Incapacitation	8	61.5
Relationship with Perpetrator		
Stranger	5	38.5
Acquaintance	7	53.8
Partner	1	7.7

Note: ^1^ Percentages do not add up to 100 due to missing data. ^2^ Participants were asked to specify if they identified as a gender other than female or male. One participant reported identifying as “other” but did not specify.

**Table 2:** Participant interview responses.

Core Theme	Sub Theme
Aesthetics and Usability	• App is simple and not overwhelming
	• Layout allows for privacy
	• Increase color brightness and font size
	• Make navigation functions clear and uniform across app
Barriers to Resources	• Logistical barriers (e.g., distance, cost, awareness of resources)
	• Attitudinal barriers (e.g., denial, perceived stigma)
Opinions about *SC-Safe*	• Education module was informative and helpful
	• Feedback on emotional and behavioral health module
	• Feedback on general coping skills

## References

[1] Walters M., Chen J., Breiding M. (2011). National Intimate Partner and Sexual Violence Survey (NISVS): 2010 Findings on Victimization by Sexual Orientation.

[2] Peterson C., Degue S., Florence C., Lokey C.N. (2017). Lifetime Economic Burden of Rape among U.S. Adults. Am. J. Prev. Med..

[3] Dworkin E.R., Menon S.V., Bystrynski J., Allen N.E. (2017). Sexual assault victimization and psychopathology: A review and meta-analysis. Clin. Psychol. Rev..

[4] Steenkamp M.M., Dickstein B.D., Salters-Pedneault K., Hofmann S., Litz B.T., Salters-Pedneault K. (2012). Trajectories of PTSD symptoms following sexual assault: Is resilience the modal outcome?. J. Trauma Stress.

[5] Office on Violence against Women (2013). National Protocol for Sexual Assault Medical Forensic Examinations.

[6] Fitzgerald K., Wooler S., Petrovic D., Crickmore J., Fortnum K., Hegarty L., Fichera C., Kuipers P. (2017). Barriers to Engagement in Acute and Post-Acute Sexual Assault Response Services: A Practice-Based Scoping Review. Int. J. Emerg. Ment. Heal. Hum. Resil..

[7] Logan T., Evans L., Stevenson E., Jordan C.E. (2005). Barriers to Services for Rural and Urban Survivors of Rape. J. Interpers. Violence.

[8] Walsh W.A., Banyard V.L., Moynihan M.M., Ward S., Cohn E.S. (2010). Disclosure and Service Use on a College Campus After an Unwanted Sexual Experience. J. Trauma Dissociation.

[9] Darnell D., Peterson R., Berliner L., Stewart T., Russo J., Whiteside L., Zatzick D. (2015). Factors Associated with Follow-Up Attendance among Rape Victims Seen in Acute Medical Care. Psychiatry.

[10] Gilmore A.K., Jaffe A.E., Hahn C.K., Ridings L.E., Gill-Hopple K., Lazenby G.B., Flanagan J.C. (2018). Intimate Partner Violence and Completion of Post-Sexual Assault Medical Forensic Examination Follow-Up Screening. J. Interpers. Violence.

[11] Ackerman D., Sugar N., Fine D., Eckert L. (2006). Sexual assault victims: Factors associated with follow-up care. Am. J. Obstet. Gynecol..

[12] Kirkner A., Relyea M., Ullman S.E. (2018). PTSD and problem drinking in relation to seeking mental health and substance use treatment among sexual assault survivors. Traumatology.

[13] Light D., Monk-Turner E. (2009). Circumstances surrounding male sexual assault and rape: Findings from the national violence against women survey. J. Interpers. Violence.

[14] Andrade L.H., Alonso J., Mneimneh Z., Wells J.E., Al-Hamzawi A., Borges G., Florescu S. (2014). Barriers to mental health treatment: Results from the WHO World Mental Health surveys. Psychol. Med..

[15] Mojtabai R., Olfson M., Sampson N.A., Jin R., Druss B., Wang P.S., Kessler R.C. (2011). Barriers to mental health treatment: Results from the National Comorbidity Survey Replication. Psychol. Med..

[16] Donne M.D., DeLuca J., Pleskach P., Bromson C., Mosley M.P., Perez E.T., Frye V. (2018). Barriers to and facilitators of help-seeking behavior among men who experience sexual violence. Am. J. Men’s Health.

[17] Turchik J.A., McLean C., Rafie S., Hoyt T., Rosen C.S., Kimerling R. (2013). Perceived barriers to care and provider gender preferences among veteran men who have experienced military sexual trauma: A qualitative analysis. Psychol. Serv..

[18] Fox S., Duggan M. (2013). Health online 2013. Health.

[19] Kilpatrick D.G., Ruggiero K.J., Acierno R., Saunders B.E., Resnick H.S., Best C.L. (2003). Violence and risk of PTSD, major depression, substance abuse/dependence, and comorbidity: Results from the National Survey of Adolescents. J. Consult. Clin. Psychol..

[20] Miller K.E., Cranston C.C., Davis J.L., Newman E., Resnick H. (2015). Psychological outcomes after a sexual assault video intervention: A randomized trial. J. Forensic Nurs..

[21] Resnick H.S., Acierno R., Amstadter A.B., Self-Brown S. (2007). An acute post-sexual assault intervention to prevent drug abuse: Updated Findings. Addict. Behav..

[22] Walsh K., McLaughlin K.A., Hamilton A., Keyes K.M. (2017). Trauma Exposure, Incident Psychiatric Disorders, and Disorder Transitions in a Longitudinal Population Representative Sample. J. Psychiatr. Res..

[23] Gilmore A.K., Flanagan J.C. (2019). Acute mental health symptoms among individuals receiving a sexual assault medical forensic exam: The role of previous intimate partner violence victimization. Arch. Women’s Ment. Heal..

[24] Hicks D.L., Patterson D., Resko S. (2017). Lessons learned from iCare: A postexamination text-messaging-based program with sexual assault patients. J. Forensic Nurs..

[25] Collins S.E., Clifasefi S.L., Loganm D.E., Samples L., Somers J., Marlatt G.A., Marlatt G.A., Witkiewitz K., Larimer M.E. (2011). Harm reduction: Current status, historical highlights and basic principles. Harm Reduction: Pragmatic Strategies for Managing High-Risk Behaviors.

[26] Nixon R.D., Best T., Wilksch S.R., Angelakis S., Beatty L.J., Weber N. (2016). Cognitive Processing Therapy for the Treatment of Acute Stress Disorder Following Sexual Assault: A Randomised Effectiveness Study. Behav. Chang..

[27] Bedard-Gilligan M., Marks E., Graham B., Garcia N., Jerud A., Zoellner L. (2016). Prolonged exposure and cognitive processing therapy for military sexual trauma-related posttraumatic stress disorder. Treating Military Sexual Trauma.

[28] Lejuez C.W., Hopko D.R., Hopko S.D. (2001). A brief behavioral activation treatment for depression. Treatment manual. Behav. Modif..

[29] Linehan M. (2015). DBT Skills Training Manual.

[30] Centers for Disease Control (2015). Sexual Assault and Abuse and STDs. https://www.cdc.gov/std/tg2015/sexual-assault.htm.

[31] Tullis T., Albert B. (2013). Measuring the User Experience: Collecting, Analyzing, And Presenting Usability Metrics.

[32] Ferney S.L., Marshall A.L., Eakin E.G., Owen N. (2009). Randomized trial of a neighborhood environment-focused physical activity website intervention. Prev. Med..

[33] Hinchliffe A., Mummery W.K. (2008). Applying usability testing techniques to improve a health promotion website. Heal. Promot. J. Aust..

[34] Leslie E., Marshall A., Owen N., Bauman A. (2005). Engagement and retention of participants in a physical activity website. Prev. Med..

[35] Stoddard J.L., Augustson E.M., Mabry P.L. (2006). The importance of usability testing in the development of an internet-based smoking cessation treatment resource. Nicotine Tob. Res..

[36] Taualii M., Bush N., Bowen D.J., Forquera R. (2010). Adaptation of a Smoking Cessation and Prevention Website for Urban American Indian/Alaska Native Youth. J. Cancer Educ..

[37] Given L.M. (2008). The Sage Encyclopedia of Qualitative Research Methods.

[38] Koss M.P., Abbey A., Campbell R., Cook S., Norris J., Testa M., Ullman S., West C., White J. (2007). Revising the SES: A Collaborative Process to Improve Assessment of Sexual Aggression and Victimization. Psychol. Women Q..

[39] Kroenke K., Spitzer R.L., Williams J.B. (2003). The Patient Health Questionnaire-2: Validity of a two-item depression screener. Med. Care.

[40] Weathers F.W., Litz B.T., Keane T.M., Palmieri P.A., Marx B.P., Schnurr P.P. (2013). The PTSD checklist for DSM-5 (PCL-5). www.ptsd.va.gov.

[41] Bovin M.J., Marx B.P., Weathers F.W., Gallagher M.W., Rodriguez P., Schnurr P.P., Keane T.M. (2016). Psychometric properties of the PTSD Checklist for Diagnostic and Statistical Manual of Mental Disorders–Fifth Edition (PCL-5) in veterans. Psychol. Assess..

[42] Wortmann J.H., Jordan A.H., Weathers F.W., Resick P.A., Dondanville K.A., Hall-Clark B., Foa E.B., Young-McCaughan S., Yarvis J.S., Hembree E.A. (2016). Psychometric analysis of the PTSD Checklist-5 (PCL-5) among treatment-seeking military service members. Psychol. Assess..

[43] Bush K., Kivlahan D.R., McDonell M.B., Fihn S.D., Bradley K.A. (1998). The AUDIT Alcohol Consumption Questions (AUDIT-C) an Effective Brief Screening Test for Problem Drinking. Arch. Intern. Med..

[44] Volk R.J., Steinbauer J.R., Cantor S.B., HOLZERIII C. (1997). The Alcohol Use Disorders Identification Test (AUDIT) as a screen for at-risk drinking in primary care patients of different racial/ethnic backgrounds. Addiction.

[45] Bradley K.A., Bush K.R., Epler A.J., Dobie D.J., Davis T.M., Sporleder J.L., Kivlahan D.R. (2003). Two brief alcohol-screening tests From the Alcohol Use Disorders Identification Test (AUDIT): Validation in a female Veterans Affairs patient population. Arch. Intern. Med..

[46] Braun V., Clarke V., Terry G., Rohleder P., Lyons A. (2014). Thematic analysis. Qualitative Research in Clinical and Health Psychology.

[47] Charmaz K. (2006). Constructing Grounded Theory: A Practical Guide Through Qualitative Research.

[48] Charmaz K. (2014). Constructing Grounded Theory.

[49] Hadfield M., Chapman A., Chapman C. (2015). Qualitative research in healthcare: An introduction to grounded theory using thematic analysis. J. R. Coll. Physicians Edinb..

[50] Hanson R.F., Gros K.S., Davidson T.M., Barr S., Cohen J., Deblinger E., Ruggiero K.J. (2014). National trainers’ perspectives on challenges to implementation of an empirically-supported mental health treatment. Adm. Policy Ment. Health Ment. Health Serv. Res..

[51] Davidson T.M., Lopez C.M., Saulson R., Borkman A.L., Soltis K., Ruggiero K.J., De Arellano M., Wingood G.M., DiClemente R.J., Danielson C.K. (2014). Development and preliminary evaluation of a behavioural HIV-prevention programme for teenage girls of Latino descent in the USA. Cult. Heal. Sex..

[52] Davidson T.M., Soltis K., Albia C.M., de Arellano M., Ruggiero K.J. (2015). Providers’ perspectives regarding the development of a web-based depression intervention for Latina/o youth. Psychol. Serv..

[53] Pelligrini A.D. (2004). Observing Children in Their Natural Worlds: A Methodological Primer.

[54] Hequembourg A.L., Parks K.A., Collins R.L., Hughes T.L. (2015). Sexual assault risks among gay and bisexual men. J. Sex Res..

[55] Black M.C., Basile K.C., Breiding M.J., Smith S.G., Walters M.L., Merrick M.T., Chen J., Stevens M.R. (2011). The National Intimate Partner and Sexual Violence Survey (NISVS): 2010 Summary Report.

[56] Alegría M., Vallas M., Pumariega A.J. (2010). Racial and Ethnic Disparities in Pediatric Mental Health. Child Adolesc. Psychiatr. Clin. North Am..

[57] Cochran S.D. (2001). Emerging issues in research on lesbians’ and gay men’s mental health: Does sexual orientation really matter?. Am. Psychol..

[58] Mayer K.H., Bradford J.B., Makadon H.J., Stall R., Goldhammer H., Landers S. (2008). Sexual and Gender Minority Health: What We Know and What Needs to Be Done. Am. J. Public Heal..

